# Combined Immunohistochemistry for the “Three 7” Markers (CK7, CD117, and Claudin-7) Is Useful in the Diagnosis of Chromophobe Renal Cell Carcinoma and for the Exclusion of Mimics: Diagnostic Experience from a Single Institution

**DOI:** 10.1155/2019/4708154

**Published:** 2019-10-13

**Authors:** Jun Zhou, Xiaoqun Yang, Luting Zhou, Peipei Zhang, Chaofu Wang

**Affiliations:** Department of Pathology, Ruijin Hospital, Shanghai Jiaotong University School of Medicine, Shanghai, China

## Abstract

**Background:**

There is a morphological overlap among renal epithelial tumors, particularly chromophobe renal cell carcinoma (CHRCC), clear cell renal cell carcinoma (CCRCC), renal oncocytoma (RO), and papillary renal cell carcinoma (PRCC). Discriminating between these tumors is important but sometimes challenging. This study is aimed at evaluating the clinical usefulness of the combined immunochemistry for the “three 7” markers (CK7, CD117, and Claudin-7) to distinguish chromophobe renal cell carcinoma from these mimics.

**Methods:**

Immunochemical staining for CK7, CD117, and Claudin-7 was performed in 68 CHRCCs, 199 CCRCCs, 32 ROs, and 30 PRCCs. Fluorescence in situ hybridization (FISH) was performed in some cases to exclude CCRCC and PRCC. The sensitivity (SE) and specificity (SP) for CHRCC as well as the immunoreactivity of each marker and their combinations were statistically evaluated.

**Results:**

High positive rates for CK7 (94%), CD117 (87%), Claudin-7 (94%), and their combinations (CK7+CD117, 79%; CK7+Claudin-7, 88%; CD117+Claudin-7, 82%; CK7+CD117+Claudin-7, 76%) were observed in CHRCC compared to those in CCRCC, RO, and PRCC, with increasingly higher SP when combinations of the “three 7” markers were applied (CK7, 0.80; CD117, 0.82; Claudin-7, 0.78; CK7+CD117, 0.95; CK7+Claudin-7, 0.97; CD117+Claudin-7, 0.97; CK7+CD117+Claudin-7, 1).

**Conclusion:**

CK7, CD117, and Claudin-7 are frequently expressed in CHRCC with high specificity. We recommend the routine use of these 3 markers as a routine panel when making a differential diagnosis of CHRCC and excluding other mimics.

## 1. Background

Chromophobe renal cell carcinoma (CHRCC) is the third most common renal cell carcinoma (RCC, 5%) and is inferior to clear cell renal cell carcinoma (CCRCC, 70-80%) and papillary renal cell carcinoma (PRCC, 15%) [1]. CHRCC is considered to have low malignant biologic behavior with a 5-year survival rate of 78-100% [2]. The somatic genomic landscape of CHRCC reveals its distal nephron origin [3]. Histologically, CHRCC is typically arranged in a sold-sheet pattern separated by a thin, incomplete, and hyalinized vascular septa [4]. Other configurations, such as nested, tubular, trabecular, cystic, alveolar, and focal papillary areas, have also been appreciated [4]. Two distinct subtypes of CHRCC have been described, that is, a typical variant and an eosinophilic variant; the classical type features a predominance of large polygonal cells with a pale and distinct cell membrane, and the eosinophilic variant demonstrates smaller cells with fine oxyphilic granularity [2, 5].

The diagnosis of renal cell carcinoma is sometimes challenging and troubling for pathologists because of the frequent histologic overlapping among each carcinoma type. The distinction of CHRCC from clear cell renal cell carcinoma (CCRCC), renal oncocytoma (RO), papillary renal cell carcinoma (PRCC), and renal cell carcinoma with XP11.2 translocation/TEF3 fusion (XP11.2 tRCC) may cause a diagnostic dilemma. Numerous immunochemical markers have been reported, including CK7, CD117 (KIT), parvalbumin, DOG1 cyclin D1, vimentin, EMA, S1001A, kidney-specific cadherin (Ksp-cad), Claudin-7, and Claudin-8 [6–9]. However, none of these markers is able to show sufficient specificity as single markers for discriminating CHRCC from other carcinomas [10]. Panels of immunostaining markers have been proposed to make a differential diagnosis: DOG1/cyclin D1/CK7/CD117/vimentin, CK7/CD117/PAX2, CK7/parvalbumin, CK7/vimentin/S100A1/CD117, S1001A/CD117, HNF1*β*/S100A1, etc. [6, 11–15]. We have 10 years of experience with the combined immunohistochemistry for the “three 7” markers, that is, CK7, CD117, and Claudin-7, to diagnose chromophobe renal cell carcinoma and exclude the mimics. This study reevaluated the sensitivity and specificity in the diagnosis of CHRCC using this “three 7” panel and described our application experience.

## 2. Methods

### 2.1. Tissue Samples

This study was approved by the Institutional Review Board of the Department of Pathology, Ruijin Hospital, Shanghai Jiaotong University School of Medicine. Cases diagnosed as CHRCC, CCRCC, RO, and PRCC with complete clinicopathologic data were selected from May 1, 2010, to May 1, 2019. All hematoxylin and eosin- (HE-) stained slides were independently reviewed by 2 experienced pathologists (X.Y. and C.F.W.). A diagnostic consensus on each case was achieved according to the 2016 *WHO Classification of Tumours of the Urinary System and Male Genital Organs* [16]. For CCRCC and PRCC, grading was assigned using the 4-tier grading system of the WHO/International Society of Urological Pathology (ISUP) [16]. In addition, PRCC and CHRCC are traditionally subcategorized into two types (PRCC: type 1 and type 2; CHRCC: classical and eosinophilic variants) according to the WHO classification [17].

### 2.2. Immunochemistry and FISH

Each surgical specimen was specifically resectioned, and the markers CD7, CD117, and Claudin-7 were stained. Four-micrometer thick sections were obtained from 10% formalin-fixed and paraffin-embedded tissue blocks, followed by immunohistochemical staining using the following commercially available antibodies: anti-CK7 (EP16, 1 : 200; ZSGB-BIO, Beijing, China), anti-CD117 (YR145, prediluted; MXB Biotech, Fuzhou, Fujian, China), and anti-Claudin-7 (polyclonal, 1 : 500; Cambridge, MA, US). Antibody binding was detected using a universal immunoperoxidase polymer method (Envision kit; Dako, Carpinteria, CA, US). A Dako automated immunohistochemistry system (Dako, Carpinteria, CA, US) was used according to the manufacturer's protocol. The IHC results were independently interpreted by 2 experienced pathologists (J.Z. and C.F.W.). More than 10% of tumor cells showing membranous or both membranous and cytoplasmic staining for CK7, CD117, and Claudin-7 were considered positive: focal, 10%-50%; diffuse, more than 50%.

For a subset of difficult cases showing overlapping morphological and immunohistochemical features, FISH was additionally applied (CCRCC, loss of chromosome 3p; PRCC, trisomy of 7 or/and 17 or loss of the Y chromosome). The procedure has been previously described [18, 19]. The probes included CEP7, CEP17, SEY (Vysis, Downers Grove, IL, USA), and CSP3+GSP 3p (LBP, Guangzhou, Guangdong, China). The signals from 100 nonoverlapping intact nuclei were counted for each lesion. Chromosome loss (or gain) was defined as the percentage of nuclei with single (or 3) signals greater than the normal tissue means for that chromosome, within 4 times the normal tissue mean for that chromosome, and within 4 times the normal standard deviation for that chromosome, as described previously [19]. Thus, for CEP7 or CEP17, the percentages of 3 or more signals of more than 10% were considered as trisomy; for CEP Y and 3p, the percentages of single signals of more than 70% and 40%, respectively, were considered as chromosome loss in this study.

### 2.3. Data Analysis

The sensitivity (SE) and specificity (SP) of CHRCC compared to those of the other 3 types of renal cell tumors (CCRCC, PRCC, and RO) were calculated using standard formulas. The differences in the immunoreactivity of each marker and their combinations were evaluated using the chi-squared test between CHRCC and CCRCC, PRCC (type 1 and type 2), or RO.

## 3. Results

**Figure 1:**
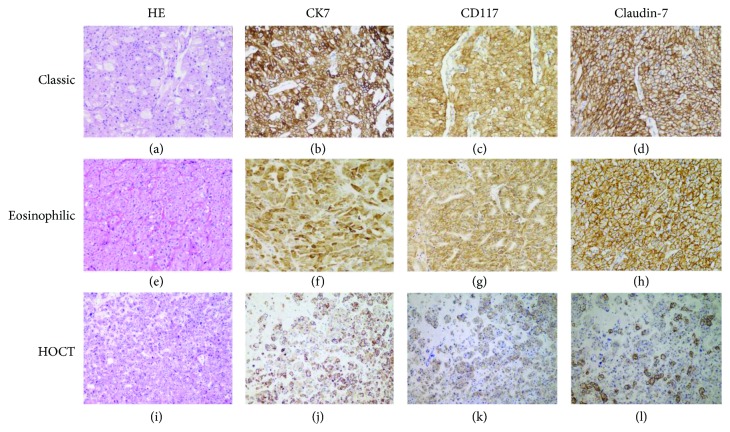
Representative expression of the “three 7” markers in 3 variants of CHRCCs. CHRCC is typically arranged in a solid figuration with delicate, incomplete fibrovascular septa containing two types of polygonal cells (chromophobe and eosinophilic cells) in a variable proportion (a); the tumor cells are diffusely and strongly positive for CK7 (b), CD117 (c), and Claudin-7 (d). The eosinophilic variant is characterized by the predominant sheets of eosinophilic cells mimicking RO (e) and is also readily highlighted by CK7 (f), CD117 (g), and Claudin-7 (h). This HOCT displays RO-like cells intimately admixed with CHRCC-like cells (i); in contrast to the negative staining in the RO-like cells, the CHRCC-like cells are typically immunoreactive for CK7 (j), CD117 (k), and Claudin-7 (l).

**Figure 2:**
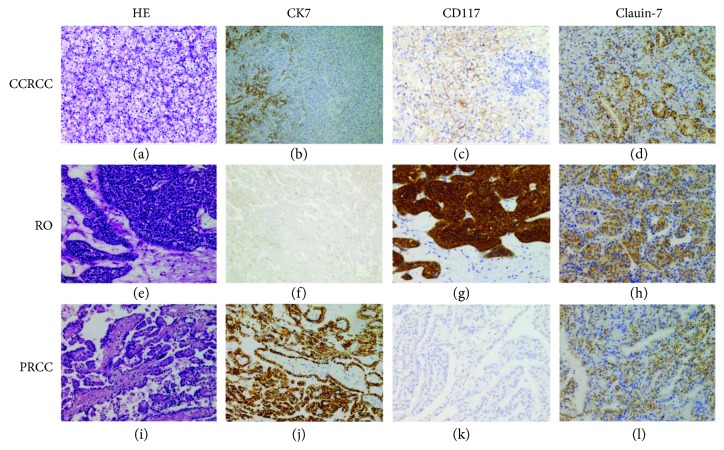
Typical expression features of the “three 7” markers in CCRCC (a), RO (e), and PRCC (i), if positive. CK7 (b), CD117 (c), and Claudin-7 (d) show focal and mild-to-moderate positivity in different cases of CCRCC. RO is typically positive for CD117 (g), but for CK7 (f) and Claudin-7 (h), the positive proportion and intensity are usually limited. Focal and moderate positivity for Claudin-7 (l) can be appreciated in PRCC; diffuse and moderate positivity for CK7 (j) is not uncommon, particularly in the type 1 PRCC, but CD117 (k) is generally negative in PRCC (note: mild positivity for Claudin-7 is usually characterized by an incompletely membranous pattern or both membranous and cytoplasmic staining patterns).

**Figure 3:**
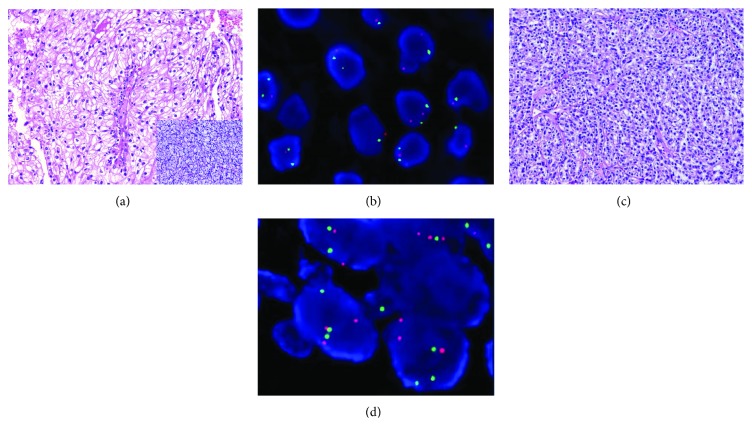
Examples for combining FISH and the “three 7” markers to confirm the diagnosis in a small subset of difficult cases. A case of CHRCC-like CCRCC (a) displays predominantly solid sheets of polygonal cells with clear to finely eosinophilic cytoplasm, a distinct membrane, and slight nuclear irregularity, mimicking CHRCC, with focal areas showing nests of clear cells with a completely delicate vascular configuration, corresponding to the morphology of CCRCC (inset of (a)); negativity for all “three 7” markers, in this case, is useless, but FISH showing the loss of 3p (b) (green signal: CSP3; red signal: 3p) facilitates a correct diagnosis in this scenario. A solid type PRCC (c) here does not show a distinct histomorphology and may occasionally mimic CHRCC or CCRCC. This rare case, only showing positivity for CK7 and focal CD117 staining (not shown), may represent a diagnostic dilemma, but FISH showing the existence of CEP7 and CEP17 trisomy (d) (green signal: CEP 7; red signal: CEP 17) without the loss of 3p contributes to the final diagnosis.

Sixty-four CHRCCs, 199 CCRCCs, 32 PRCCs, and 30 ROs were eventually included in our analysis. The 68 CHRCCs contained 55 classical (Figure 1(a)), 5 hybrid oncocytic/chromophobe (HOCT; Figure 1(i)), and 8 eosinophilic (Figure 1(e)) variants, and one case of the latter had a focal area of sarcomatoid change. HOCT was newly included in the 2016 WHO classification, with an overlapping morphology between CHRCC and RO [2]. The WHO/ISUP classification of 199 CCRCCs (Figure 2(a)) was grade 1 (12%; *n* = 24), grade 2 (63%; *n* = 125), grade 3 (19%; *n* = 38), and grade 4 (6%; *n* = 12). Grading was not performed in ROs (Figure 2(e)) due to lack of established evaluating system. The 32 PRCCs (Figure 2(i)) included 9 type 1 (28%), 21 type 2 (66%), and 2 solid PRCCs (6%), and the WHO/ISUP classification was grade 1 (9%; *n* = 3), grade 2 (44%; *n* = 14), grade 3 (44%; *n* = 14), and grade 4 (3%; *n* = 1). To confirm the separation of a subset of renal tumors, the gene status of 3p (*n* = 10) or the combinations of chromosomes 7, 17, and Y for PRCC (*n* = 14) were detected using FISH. All CCRCCs showed a loss of 3p (3/3, Figures 3(a) and 3(b)); most of the PRCC (8/11) samples had at least one abnormality involving chromosomes 7, 17, or Y (trisomy of 7, 7/11; trisomy of 17, 6/11; loss of chromosome Y, 4/6; Figures 3(c) and 3(d)); and no CHRCC (0/3) samples showed these genetic perturbations.

**Figure 4:**
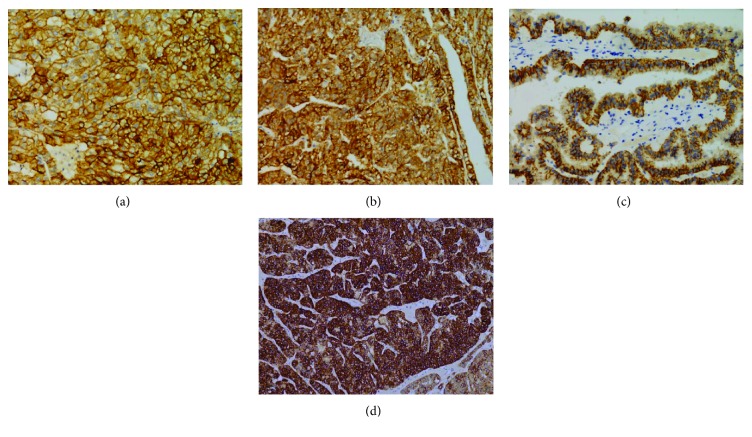
Exceptional cases with diffuse and strong immunostaining for some of the “three 7” markers in CCRCC and PRCC. Herein, 2 distinct cases of CCRCC show bright CK7 (a) and Claudin-7 (b) immunostaining, respectively. Rare cases of PRCC can show diffuse and strong positivity for Claudin-7 (c). Both CCRCC and PRCC are consistently negative for CD117. This rare case of RO shows diffuse and intense Claudin-7 (d) expression.

**Figure 5:**
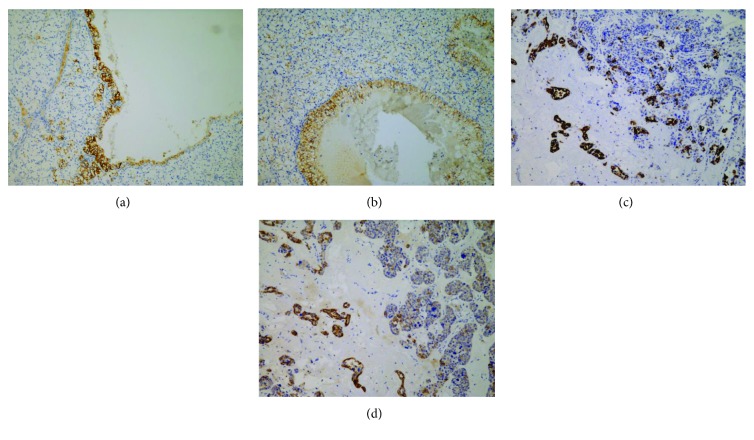
Miscellaneous immunohistochemical features of CK7 and Claudin-7 in some negative cases of CCRCC and RO. Very focal (<10%) and mild-to-moderate staining for CK7 or Claudin-7 can be found in some tumor cells located on the wall of cystic structures (CCRCC: (a) CK7; (b) Claudin-7) or within sclerotic/edematous stroma (RO: (c) CK7; (d) Claudin-7) in some “three 7”-negative cases of CCRCC or RO.

The immunohistochemical results are summarized in Table 1. The majority of CHRCCs expressed CK7 (94%; 64/68), CD117 (87%; 59/68), and Claudin-7 (94%; 64/68), and the positive proportion of the combination of these 3 markers was 79% (CK7+CD117; 54/68), 88% (CK7+Claudin-7; 60/68), 82% (CD117+Claudin-7; 56/68), and 76% (CK7+CD117+Claudin-7; 52/68) (Figure 1). Most CHRCCs showed diffuse and strong positive staining for CK7 (95%; 61/64; Figures 1(b), 1(f), and 1(j)), CD117 (95%; 61/64; Figures 1(c), 1(g), and 1(k)), and Claudin-7 (97%; 62/64; Figures 1(d), 1(h), and 1(l)). Five HOCTs (Figure 1(i)) displayed prototypical mixed immunophenotypes: CHRCC-like area strongly and diffusely expressing CK7, CD117, and Claudin-7, whereas RO-like areas were negative for CK7 and Claudin-7 but not CD117 (Figures 1(j)–1(l)). For CCRCC (Figure 2(a)), 8% (16/199), 0.5% (1/199), and 12% (24/199) of cases were immunoreactive for CK7 (focal, 13/16, Figure 2(b); diffuse, 3/13, Figure 4(a)), CD117 (focal, 1/1; Figure 2(c)), and Claudin-7 (focal, 6/24, Figure 2(d); diffuse, 18/24, Figure 4(b)), respectively. The combinations of these 3 markers were minimally stained: CK7+CD117 (0/199), CK7+Claudin-7 (3/199), CK7+Claudin-7 (0/199), and CK7+CD117+Claudin-7 (0/199). In ROs (Figure 2(e)), slight CK7 (7%; 2/30; Figure 2(f)), Claudin-1 (27%; 8/30; Figure 2(h)), CK7+CD117 (3%; 1/30), and CD117+Claudin-7 (17%; 5/30) staining and strong CD117 (83%; 25/30; Figure 2(g)) staining were observed. None of the “CK7+Claudin-7” (0/30) and “CK7+CD117+Claudin-7” (0/30) combinations was positive in ROs. As for PRCC (Figure 2(i)), 47% (15/32) of cases showed positivity for CK7 (focal, 3/15; diffuse, 12/15; Figure 2(j)), 16% (5/32) for CD117 (focal, 4/5; diffuse, 1/5; Figure 2(k)), and 38% (12/32) for Claudin-7 (focal, 2/12; diffuse, 10/12; Figure 2(l)). Among the positive results, more type 1 PRCCs displayed positivity for CK7 (89%; 8/9), CD117 (22%; 2/9), Claudin-7 (78%; 7/9; Figure 4(c)), CK7+CD117 (22%; 2/9), and CK7+Claudin-7 (67%; 6/9) than did type 2 PRCCs with 27% CD7 (6/22), 14% CD117 (3/22), 18% Claudin-7 (4/22), 9% “CK7+CD117” (2/22), and 9% “CK7+Claudin-7” (2/22) expression. Both types were totally negative for the “CD117+Claudin-7” (type 1, 0/9; type 2, 0/22) and “CK7+CD117+Claudin-7” (type 1, 0/9; type 2, 0/22) combinations. All positive staining in PRCCs was mild to moderate compared to that in CHRCCs. The staining intensity for almost all 3 markers in CCRCC, PRCC, and RO (only 1 case was strongly positive for Claudin-7, Figure 4(d)) was mild to moderate compared to that in CHRCC. For a negative subset of CCRCCs and ROs, very focal (<10%) and mild-to-moderate staining for CK7 (Figures 5(a) and 5(c)) or Claudin-7 (Figures 5(b) and 5(d)) was also appreciated in some tumor cells that are usually located on the wall of cystic structures or within sclerotic/edematous stroma.

The SE and SP of the “three 7” markers in CHRCC and comparisons of CHRCC with CCRCC, PRCC, and RO are summarized in Table 2. The SE and SP of CK7 in CHRCC were 0.91 and 0.80, respectively. Claudin-7 showed higher SE and slightly lower SP (SP = 0.78) than those of CK7. The SP of CD117 was 0.82, while the SE (SE = 0.94) was relatively higher than that of CK7. The SP for the combination of the 3 markers was significantly enhanced, but the SE was consequently decreased. The results are shown in descending order of SP as follows: CK7+CD117+Claudin-7 (SP = 1; SE = 0.76); CD117+Claudin-7 (SP = 0.97; SE = 0.85) and CK7+Claudin-7 (SP = 0.97; SE = 0.81); and CK7+CD117 (SP = 0.95; SE = 0.81). The expression of CK7, CD117, Claudin-7, and their combinations was significant in CHRCC when compared to CCRCC, type 1 PRCC, type 2 PRCC, and RO (*p* < 0.0001; *p* = 0.0003 for CK7+CD117 in CHRCC vs. PRCC, type 1), except for the expression of CK7 or Claudin-7 between CHRCC and type 1 PRCC and CD117 between CHRCC and RO.

## 4. Discussion

There are overlapping morphological features among CHRCC, PRCC, CCRCC, and RO, often leading to diagnostic challenges when encountering difficult cases. Immunohistochemical staining, compared to electron microscopy or Hale colloidal iron staining, is an easier way to facilitate the discrimination of these carcinomas [20]. Although a collection of immunostaining markers and/or morphologic features have been described, none of these molecules is pathognomonic. Similarly, various markers and their combinations have been applied to differentiate CHRCC and other mimics, but none of these proteins is absolutely specific to the diagnosis of CHRCC [6, 8, 11, 13, 21].

Notably, 60-100% of CHRCCs are positive for CK7, with a typically diffuse (90-100% of tumor cells) and strong staining pattern [21, 22]. Consistently, our study showed high SE for CK7 (94%) in CHRCC but relatively lower SP (0.87) than that in CCRCC, PRCC, and RO. The proportion of negative cases may lead to diagnostic dilemmas if one relies only on CK7 and histomorphology. Only limited CCRCCs and ROs are positive for CK7, and even if positive, these tumors usually demonstrate a focal pattern with mild-to-moderate intensity. In contrast to type 2 PRCC, CK7 is frequently labeled in type 1 carcinomas, suggesting that this marker is useless for differential diagnosis (*p* = 0.5497). Some negative cases can show marked focal positivity for CK7 in either CCRCC or RO [23]. The scattered expression of CK7 can be observed in some cells of high-grade tumors or the lining of cystic walls in CCRCC [24]. According to our experience, CK7 may also be positive in the clear cells of CCRCCs with a superimposed tall/columnar or eosinophilic appearance, but these areas are frequently very limited (usually <10%). CD117 labels both the majority of CHRCCs and ROs, commonly with strong and diffuse staining patterns [25]. The SP of CK7 in CHRCC should be higher than that in RO, which is not consistent with the results in our study (CK7 vs. CD117, 0.87 vs. 0.88) because the proportion of RO cases was significantly less than that of other CK7-negative renal cell carcinomas (particularly, CCRCC). Therefore, CK7 is useless for differentiation from ROs intimately mimicking CHRCCs only by the expression of CD117, but this molecule can facilitate the separation of CCRCC (0.5%) from PRCC (16%). The latter two demonstrate focal and mild-to-moderate positivity, even if these carcinomas express CD117. Previous gene expression microarray analysis revealed that Claudin-7 was overexpressed in CHRCC versus oncocytoma and other tumor subtypes [26]. Analogous to the expression of CK7, Claudin-7 usually shows diffuse positivity in the majority of CHRCCs (80%-100%) [27–30]. Although a subset of CCRCCs (0-26.1%), PRCCs (28-90%), and OCs (26%-81.8%) can be reactive for Claudin-7 [26, 28–31], the positive results of Claudin-7 expression were variable, with a wide range among different research groups. The main reason for this discrepancy is likely the different criteria for the interpretation of Claudin-7 positivity. In our case series, only Claudin-7 reactivity greater than 10% was considered positive. Claudin-7 usually shows mild-to-moderate cytoplasmic reactivity and mild and discontinuous membranous reactivity in some renal cell carcinomas, all of which should not be counted as positive staining in diagnostic practice. Although a subset of CCRCCs, PRCCs, and ROs express Claudin-7, most of these carcinomas demonstrate mild or moderate membranous positive staining. In contrast, strong membranous or both membranous and cytoplasmic reactivity for Claudin-7 is the representative staining in CHRCC, similar to the expression of Her-2 with a score of 3+ in mammary invasive carcinoma. In addition, we found that Claudin-7 is usually positive in fibrotic/hyaline, edematous, or cystic regions in renal cell carcinomas, regardless of the reactivity of the tumor for Claudin-7. This very focal or mild cytoplasmic positivity is basically useless for the diagnosis of CHRCC if considered positive staining. In addition, positive Claudin-7 staining is not uncommon in type 1 PRCC, and therefore, there is limited discrimination between CHRCCs and type 1 PRCCs.

The recently described HOCTs have been observed in distinct clinical settings, including renal oncocytosis, Hogg–Dubé syndrome, or sporadic RCC [32]. HOCTs are characteristic of a mixture of RO-like and CHRCC-like tumor cells, either as distinctly separate tumor groups adjacent to one another, intimately admixed with one another, or showing a gradual transition from one typical region to another [33]. Although HOCT, as a variant, is under the umbrella of CHRCC, increasing evidence shows different genomic features supporting its distinct nature from CHRCC [34]. Nevertheless, the distinct components of HOCT still adhere to the expression pattern of the “three 7” markers observed in their corresponding renal tumors. These different expression modes essentially contribute to the diagnosis of HOCTs.

Although there were papers applying CK7, CD117, or Claudin-7 with other markers to make differential diagnoses, the combination of these 3 markers has never been reported.

In diagnostic practice, differential diagnoses of CHRCC, PRCC, CCRCC, and RO are routinely performed before the given diagnosis of any of these 4 tumors is made. In this scenario, the specificity of immunostaining markers is a priority but the sensitivity is secondary. The diagnostic SP for CHRCC of the combination of any two of the markers CK7, CD117, and Claudin-7 was significantly enhanced compared to that of a single immunostaining marker. The application of these “three 7” markers together reached 100% SP in our study. In the past 10 years of practice, we have routinely used these 3 markers when differentiating CHRCC from other renal cell carcinomas. When histologic overlapping exists between CHRCC and other renal cell carcinomas, more than 2 positive markers are more likely to support the diagnosis of CHRCC (SP, 0.95-1). If only one or none of the 3 markers was positive, then the diagnosis of CHRCC is suggestive but uncertain, and therefore, further investigations should be carried out. We usually perform special staining colloidal iron and FISH tests (CEP7, CEP17, SEY, and CSP3+GSP 3p) to exclude the other mimics. However, morphology is a priority, when there is a paradox between the typical histological features of a given carcinoma and the immunohistochemical staining. Cases that do not benefit from “three 7” immunostaining (only 1 or no marker positive) without superimposed classical morphology and distinct molecular markers present truly difficult situations, and most of these cases are designated as unclassified renal cell carcinomas. However, additional markers need to be investigated in the future.

## 5. Conclusions

In conclusion, our study and experience demonstrated that CK7, CD117, and Claudin-7 are frequently expressed in CHRCC with high specificity. Knowing the expression features and patterns facilitates the interpretation of positive staining in the renal cell carcinomas. Application of the “three 7” markers, if necessary, with some special staining and/or molecular tests can resolve a majority of the diagnostic issues for CHRCC. We recommend that these 3 markers are used as a routine panel in the differential diagnosis of CHRCCs from other mimics.

## Figures and Tables

**Table 1:** Immunohistochemistry results of the “three 7” markers in CHRCC, CCRCC, PRCC, and OC.

Markers	Results	CHRCC *n* (%)	CCRCC *n* (%)	PRCC *n* (%)	OC *n* (%)
CK7	+	64 (94)	16 (8)	15 (47)	2 (7)
	−	4 (6)	183 (92)	17 (53)	28 (93)

CD117	+	59 (87)	1 (0.5)	5 (16)	25 (83)
	−	9 (13)	198 (99.5)	27 (84)	5 (17)

Claudin-7	+	64 (94)	24 (12)	12 (38)	8 (27)
	−	4 (6)	175 (88)	20 (62)	22 (73)

CK7+CD117	+	54 (79)	0 (0)	4 (13)	1 (3)
	−^∗^	14 (21)	199 (100)	28 (87)	29 (97)

CK7+Claudin-7	+	60 (88)	3 (1.5)	9 (28)	0 (0)
	−^∗^	8 (12)	196 (98.5)	23 (72)	30 (100)

CD117+Claudin-7	+	56 (82)	0 (0)	0 (0)	5 (17)
	−^∗^	12 (18)	199 (100)	32 (100)	25 (83)

CK7+CD117+Claudin-7	+	52 (76)	0 (0)	0 (0)	0 (0)
	−^∗^	16 (24)	199 (100)	32 (100)	30 (100)

^∗^Defined as negative when one of any marker was not positive.

**Table 2:** Sensitivity and specificity of the “three 7” markers in CHRCC and comparisons of CHRCC with CCRCC, PRCC, and OC.

Markers	Sensitivity	Specificity	CHRCC vs. CCRCC *p* value	CHRCC vs. PRCC, type 1 *p* value	CHRCC vs. PRCC, type 2 *p* value	CHRCC vs. OC *p* value
CK7	0.91	0.80	<0.0001	0.5497	<0.0001	<0.0001
CD117	0.94	0.82	<0.0001	<0.0001	<0.0001	0.6546
Claudin-7	0.94	0.78	<0.0001	0.0857	<0.0001	<0.0001
CK7+CD117	0.81	0.95	<0.0001	0.0003	<0.0001	<0.0001
CK7+Claudin-7	0.81	0.97	<0.0001	<0.0001	<0.0001	<0.0001
CD117+Claudin-7	0.85	0.97	<0.0001	<0.0001	<0.0001	<0.0001
CK7+CD117+Claudin-7	0.75	1	<0.0001	<0.0001	<0.0001	<0.0001

## Data Availability

The datasets generated during and/or analysed during the current study are available from the corresponding authors on reasonable request.
